# Automated seizure diagnosis system based on feature extraction and channel selection using EEG signals

**DOI:** 10.1186/s40708-021-00123-7

**Published:** 2021-02-12

**Authors:** Athar A. Ein Shoka, Monagi H. Alkinani, A. S. El-Sherbeny, Ayman El-Sayed, Mohamed M. Dessouky

**Affiliations:** 1 Department of Computer Science and Engineering, Faculty of Electronic Engineering, Menoufia University, Menouf, Egypt; 2 Department of Computer Science and Artificial Intelligence, College of Computer Science and Engineering, University of Jeddah, Jeddah, Saudi Arabia; 3 Department of Industrial Electronics and Control Engineering, Faculty of Electronic Engineering, Menoufia University, Menouf, Egypt

**Keywords:** Seizure, Epilepsy, Electroencephalography (EEG), Feature extraction, Channel selection, Cross-validation, And seizure classification

## Abstract

Seizure is an abnormal electrical activity of the brain. Neurologists can diagnose the seizure using several methods such as neurological examination, blood tests, computerized tomography (CT), magnetic resonance imaging (MRI) and electroencephalogram (EEG). Medical data, such as the EEG signal, usually includes a number of features and attributes that do not contains important information. This paper proposes an automatic seizure classification system based on extracting the most significant EEG features for seizure diagnosis. The proposed algorithm consists of five steps. The first step is the channel selection to minimize dimensionality by selecting the most affected channels using the variance parameter. The second step is the feature extraction to extract the most relevant features, 11 features, from the selected channels. The third step is to average the 11 features extracted from each channel. Next, the fourth step is the classification of the average features using the classification step. Finally, cross-validation and testing the proposed algorithm by dividing the dataset into training and testing sets. This paper presents a comparative study of seven classifiers. These classifiers were tested using two different methods: random case testing and continuous case testing. In the random case process, the KNN classifier had greater precision, specificity, positive predictability than the other classifiers. Still, the ensemble classifier had a higher sensitivity and a lower miss-rate (2.3%) than the other classifiers. For the continuous case test method, the ensemble classifier had higher metric parameters than the other classifiers. In addition, the ensemble classifier was able to detect all seizure cases without any mistake.

## Introduction

Epilepsy is a central nervous system condition (neurological) that causes irregular brain function, seizures or periods of strange behavior, feeling and often loss of consciousness. Seizure symptoms may vary greatly. Some people with seizures simply look blankly for a few moments during a seizure, while others constantly move their arms or legs. Having a single seizure does not mean you have epilepsy. The diagnosis of epilepsy usually involves at least two ineffective seizures.

Neurologists can diagnose the seizure using several methods such as neurological examination, blood testing, electroencephalogram (EEG), computerized tomography (CT), magnetic resonance imaging (MRI), positron emission tomography (PET), and single-photon emission computerized tomography (SPECT) [1]

- *Neurological exam*, focuses on the patient’s actions, brain skills and mental activity to assess the patient’s brain and nervous system.
- *Blood test*, tracks the symptoms of infection, medical defects and levels of blood sugar.
- *Electroencephalogram *(*EEG*)* test*, the neurologist sets electrodes to the patient’s head using a paste-like material. The electrical activity of the brain will be reported by the electrodes.
- *Computerized tomography *(*CT*)* scans*, can show anomalies in the patient’s brain that may cause seizures, including tumors, bleeding and cysts.
- *Magnetic resonance imaging *(*MRI*)* test*, detects lesions or defects in the brain of the patient that may induce seizures.
- *Positron emission tomography *(*PET*)* scans*, help represent the brain's active areas and detect anomalies.
- *Single-photon emission computerized tomography *(*SPECT*), can provide even more accurate results.

Early Seizure detection is critical in the medical field. Humans are undergoing various kinds of stress in their daily lives, and many of them are suffering from different neurological disorders. The World Health Organization (WHO) has announced that epilepsy is one of the most common diseases of nearly 50 million people worldwide, with more than 75% living in developing countries with little or no access to scientific services or treatment [2]. Recurrent seizures related to sudden sporadic neuronal releases in the cerebrum are described as epilepsy [3, 4].

Epileptic seizures are one of the most common diseases in the central nervous system. It results from sudden and unexpected electrical disturbances of the brain or electrical discharge caused by a group of brain cells. Individuals suffering from epilepsy have different symptoms, such as unusual sensations, twitching of arms, vision changes, hearing, smelling, or unexpectedly seeing things so that they are unable to perform regular tasks. Although; usually patients do not have any physical symptoms [5, 6].

Epilepsy leads to shivering and sudden movements, and even causes patients to lose their lives. It is therefore exceptionally crucial for the accurate automatic detection of epileptic seizures. Epilepsy requires a reliable and accurate strategy to predict seizure events to make the lives of patients less complicated [7, 8].

The main contribution of this work can be summarized as follows:

1. Building an automated Seizure detection system based on classifying the most significant extracted features using EEG signals.
2. Selecting the most affected channels from the CHB-MIT EEG signal dataset [9] and extracting the most relevant features from the selected channels.
3. Measuring the performance evaluation of seven classifiers by the CHB-MIT dataset.
4. Testing the seven classifiers using cross-validation.
5. Calculating accuracy, sensitivity, specificity, F1-score, FallOut, and MisRate of performance metric parameters for seven classifiers.

The proposed model is based on machine learning approach to achieve the objectives of this study. The main objective of this paper is to automatically Seizure detection by extracting the most significant features from the CHB-MIT EEG signal dataset and classifying the EEG signal weather it is Seizure or normal using seven classifiers.

This paper is organized as follows: Sect. 2 presents the previous and related work. Section 3 provides a description of the proposed algorithm. The EEG signal data set is described in Sect. 4. Section 5 lists the evaluation of performance metrics. The results will be presented in Sect. 6. The end of the paper is the conclusion and the references.

## Related work

Electroencephalography (EEG) is one of the primary modalities regularly used for remote epileptic seizure detection. It had become an inexpensive and non-invasive stage to investigate the inconspicuous quality of the disease. Seizure is a characterizing property of epilepsy that reflects abnormal periods of activity in the EEG [10].

Machine learning (ML) is the fastest growing field in the field of computer science and, in particular, in the field of health informatics. ML’s goal is to develop algorithms that can learn and improve over time and can be used for predictions. The overall aim is to build and develop algorithms that can automatically learn from data and therefore enhance with experience over time without any human-in-the-loop technology [11].

ML is a very realistic field of AI with the purpose of producing software which can automatically learn from existing data to learn from experience and keep improving its learning decisions to make assumptions due to new data. ML can be used as an AI workhorse, and meanwhile, the deployment of data-intensive ML algorithms could be observed all over everything, across science, engineering and business, resulting to much more evidence-based decision-making. There is a massive market for AI algorithms in medicine, that not only execute excellently, but are reliable, consistent, easy to interpret and understandable to a personal knowledge; in medicine [12].

Explaining of AI may greatly improve the confidence of healthcare experts in future AI systems. Explaining designing research-AI systems for use in medicine need a high level of learning ability across a variety of ML and human–computer interaction methods. There is an intrinsic discrepancy between the output of ML (predictive accuracy) and the ability to clarify. Mostly the best-performing strategies are the least straightforward, and those that offer a simple description are less reliable [12].

Several algorithms were designed for early diagnosis of epilepsy using an EEG signal. The problem here is that a number of features may be irrelevant and dispensable. For example, different algorithms were used to extract critical features such as auto-regressive (AR) [13], principle component analysis (PCA) [14], empirical mode decomposition (EMD) [15], and statistical features technique [16]. There is another method of extracting a statistical feature that has been widely used to extract features in several algorithms to improve performance [17, 18].

The EEG signal consists of multi-channel signals that carry a lot of repetitive data, with an additional source of noise that may reduce the accuracy of the classification. Channel selection is an important step that effectively avoids redundant channels, eliminates calculations, particularly in real-time applications, and selects the ideal classification channels. Channel selection is a significant method for reducing the number of channels, not including distinguishing information, and also for reducing noise [19, 20].

Several algorithms have used the concept of channel selection with different types. One algorithm combined the advantages of both feature enhancement and channel selection to advance the performance of the detector [21]. Another algorithm compared the reduction of electrode mounting using only nine electrodes instead of all 23 electrodes [22]. Various algorithms selected EEG channels to eliminate power consumption in the detection process without affecting accuracy, variance, variance difference, entropy, random selection and extra focal channels, as well as physician choice, are also used and are the result of valid selection. Variance is one of the most commonly used channels [23, 24].

This paper focuses on the collection and extraction of features widely used in a number of previous work. These features include standard deviation, mean, variance, median, kurtosis, skewness, entropy, moment, power, maximum and minimum EEG signals. All of those features are divided into three categories: the first category is statistical features such as mean, standard deviation, variance, skewness, median and kurtosis. The second category is amplitude-related features that include energy, power, maximum and minimum EEG signals and the third category is entropy-related feature. These features can be classified on the basis of their description or the field where the attributes are determined. Many other researchers have found a basic group of attributes appropriate to their suggested classification system, while some have introduced different groups of variables obtained from time, frequency and time–frequency domains [25–30]

Classification is the process of identifying groups or classes based on similarities between them. This step is essential to distinguish between seizure itself—the ictal period—and the normal non-ictal period. Several algorithms have been used as a classifier such as artificial neural network (ANN) [31], support vector machine (SVM) [32], ensemble [33], K-nearest neighbors (KNN) [34, 35], linear discriminant analysis (LDA) [36, 37], logistic regression [38], decision tree [39], and Naïve Bayes [40, 41].

Several previous algorithms used only one classifier to classify seizure activity from the EEG signal. Others used more than one algorithm and compare between the results of each classifier. In [31], the authors extracted 12 features from the EEG seizure signal and entered them into four different classifiers; artificial neural network (ANN), least square-support vector machine (LS-SVM), random forest and Naïve Bayes. In [32], the authors classified the EEG seizure signal using two levels of classifier. The first level is the SVM classifier, the second level is Naive Bayes classifier. The ensemble algorithm had been used for the seizure classification in [33]. In [37], the authors compared the results of three classifiers; quadratic discriminant analysis (QDA), K-nearest neighbors (KNN), and linear discriminant analysis (LDA) algorithms to classify the EEG seizure signals. In [38], the authors first extracted the significant seizure features from the EEG signal using wavelet transform, then these features had been classified using artificial neural network (ANN) and logistic regression (LR). The decision tree classifier had been used for the classification of epilepsy in [38].

The cross-validation step is an essential step before classification, which provides an accurate indication of the performance of the classifier. Cross-validation to divide the extracted features into training and test sets. In the first step, dataset information or concepts are grouped into two classes (seizure and normal) for model learning. The second step, the model of the preceding step, is used for classification [42].

The efficiency of the proposed seizure diagnostic system was measured by calculating multiple output metric parameters. Numerous previous models tested the effects of their algorithm by measuring the precision, sensitivity, specificity and computational complexity that were calculated as relative values using the computational cost needed to produce each feature [25]. Several performance metric parameters were calculated in this paper, such as precision, sensitivity, specificity, F1-score, FallOut, and misRate.

## Proposed approach

The main objective of this paper is to automatically predict epileptic seizures from EEG signals. EEG records ictal and non-ictal cases, the proposed approach detects each seizure state, and it is acceptable to classify the normal seizure state in limited cases, and this ensures that any complications are avoided. The opposite case, however, is not acceptable.

**Fig. 1:**
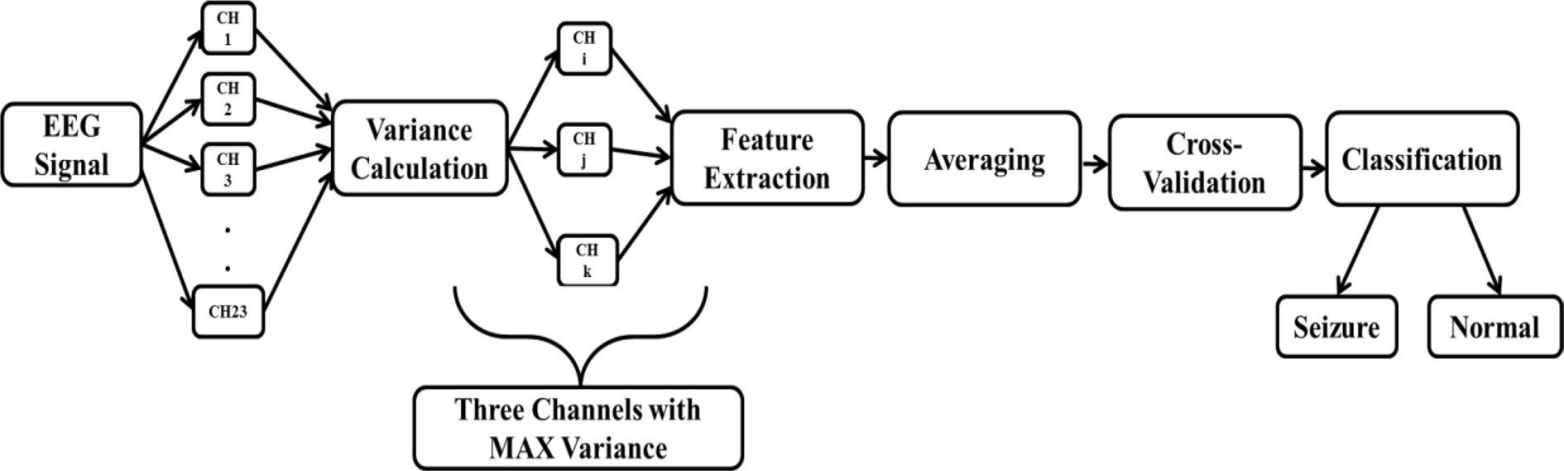
Block diagram shows the steps of the proposed algorithm

Essentially, the proposed approach relies on the recognition of epilepsy from a short-term EEG signal. The proposed approach consists of five stages: channel selection, feature extraction, average, cross-validation and classification. All of these steps are shown in Fig. 1.

The EEG signal consists of 23 channels generated by the electrodes which are attached to the scalp. These channels make the calculations more complex and increase the system load. Due to these limitations, the channel selection step is very important. These selected channels will be used as input to the extraction step of the feature. The third step is the average where the features extracted from the selected channels will be averaged. Finally, the average features will be used as the classifier input. Several performance metric parameters were measured to evaluate the performance of the seven classifiers to compare them, one of which has improved performance.

The main steps of the proposed algorithm are:

1. *Variance channel selection* which used for dimensionality reduction by selecting the most affected channels using the variance parameter.
2. *Feature extraction and averaging*, which used to extract the most significant features, eleven features, from the selected channels. Then, the averaging of these extracted features from each channel is added.
3. *Classifying* the averaged features for distinguishing between normal and seizures signals to better detect and diagnose seizures. This move is to classify groups or classes based on similarities between them. This phase is important to distinguish between the Seizure itself—the ictal stage—and the usual non-ictal era.
4. *Cross-validation* to divide the extracted features from the CHB-MIT EEG signal dataset [9] into training and testing sets. This step is an important step which gives a precise indication of the performance of the classifier. Cross-validation consists of two stages; training and testing. During the training process, the dataset information is grouped into two classes (seizure and normal) to learn the model. During the testing process, the trained model is used to assess new signals to identify them as seizure or normal.
5. *Performance evaluation* of proposed approach with existing algorithms. In this step, the proposed approach is applied to two separate methods; the random circumstance testing and the continuous circumstances to measure the accuracy, sensitivity, specificity, F1-score, FallOut, and misRate of the proposed approach.

### Variance channel selection

The channel selection step is designed to select the most affected channels by seizure. Variance is chosen as the channel selection method, because experiments show that automatic seizure detection can be performed using only three channels, selected on the basis of maximum variance without loss of performance. The variance is used to calculate for all channels, according to this feature, the channel would be selected, and then the other features would be calculated for the selected channels only.

This step is essential to reduce processing load and time. Each channel generates 11 features, so the total input nodes for each model would be 11 × 23 channels that make the calculations take a long time in training and testing.

The simple method for selecting channels for extraction and classification features is the variance of the EEG signal amplitude, since automatic seizure detection can be performed using only three channels without loss of performance. These channels are selected on the basis of the maximum variance.

The variance (*V*) of the sample I in channel (*c*) of the training data (*t*) is calculated in Eq. (1) [24]:

1 $${V}_{ict}\left(c\right)=\frac{1}{k}\sum_{i=1}^{k}({X}_{c}\left(i\right)-{\mu }_{c})2,$$

where $c$ is the channel, ${X}_{\mathrm{c}}$ is the data on seizure training, ${\mu }_{\mathrm{c}}$ is the mean of seizure training data, *k* is the number of samples of seizure training data.

The selection of channels based on the highest values of ${V}_{ict}(c)$ is calculated using Eq. (2) [24]:

2 $$\mathrm{chosen}\, \mathrm{channel}=\mathrm{max}({V}_{ict}(c))$$

At the end of this step, the highest three channels with maximum variance are selected to extract the significant features from only those three channels.

### Feature extraction and averaging

Feature extraction is a specific form of dimensional reduction. Feature extraction is a general term for the methods used to construct a combination of variables. In this step, some distinctive features will be extracted from the selected EEG signal channels. These features have the most influence in the form of a signal. These features are extracted from the EEG signal in ten seconds. Various types of features can be extracted from the EEG signal.

These features are the standard deviation, mean, variance, median, kurtosis, skewness, entropy, moment, power, maximum and minimum EEG signals defined as [17, 18, 30]:

1. *Standard deviation*: is the mean value of the EEG signal and is calculated from the equation, where *D* is the signal and *N* is the number of samples *µ* is the square root of the variance.
  3 $$\sigma =\sqrt{\frac{1}{N-1}{\sum }_{i=1}^{N}({D}_{i}-\mu }{)}^{2}$$
2. *Mean*: is the basic statistical and calculated from the following equation, where $i=1, 2, 3,\dots$ and *D* is the signal.
  4 $${\mu }_{i}=\frac{1}{N}\sum_{j=1}^{N}{D}_{ij}.$$
3. *Variance*: is obtained by taking the standard deviation square.
  5 $$v={\sigma }^{2}$$
4. *Median*: is a simple measure of the central tendency and is calculated from the equation below.
  6 $$\stackrel{-}{X}=\frac{{\sum }_{i=1}^{n}{x}_{i}}{n}$$
5. *Kurtosis*: measures the height of the probability density function (PDF) of the time series.
  7 $$k=\frac{E(x-\mu {)}^{4}}{{\sigma }^{4}}$$
6. *Skewness*: represents the PDF symmetry of the amplitude of the time series.
  8 $$s=\frac{E(x-\mu {)}^{4}}{{\sigma }^{4}}.$$
7. *Entropy*: is the numerical proportion of the arbitrary nature of the signal.
  9 $$E\left(s\right)={\sum }_{i}E({s}_{i})$$
8. *Moment*
  10 $$m=\mathrm{moment }\left(x.\mathrm{ order}\right)$$
9. *Maximum EEG signal*: returns the max point in the signal.
  11 $$M= \mathit{max} (D)$$
10. *Minimum EEG signal*: returns the min point in the signal.
  12 $$M=\mathrm{min }(D)$$
11. *EEG signal power*: is calculated from these equations.
  13 $$f=fft(s)$$
  14 $$\mathrm{pow}=\mathrm{sum }\left({f}^{\mathrm{^{\prime}}}*\mathrm{conj}\left(f\right)\right).$$

Each of the three channels selected produces 11 features, so that the input for each model would be 11 × 3 for each case, which would affect the calculations in real time and could prolong the classification time. Averaging the values of the extracted features would reduce the number of input nodes of the model and eliminate the processing load and the time of classification [8].

### Classification

Classification is a technique in which the data is classified into a set of classes. The key purpose of the classification is to classify the class to which the data would belong. Classification algorithms are divided into supervised, unsupervised and semi-supervised algorithms. Supervised algorithms are based on training and testing the data. The trained data are labelled and the labels will be sent to the model through implementation. This labeled dataset is trained to produce significant outputs as it is processed by decision-making. Unsupervised algorithms are based on data classification without the training of the classifier. It’s not a genre, there’s no history, no training, and no data testing. They do not give the right goals and instead depend on clustering. Semi-supervised algorithms are mixed of supervised and unsupervised algorithms. So, some data are labelled and others are not labelled. Algorithms may be applied to labeled and unmarked data, and some dataset classifiers will be learned for either complete information or missing training sets.

Seven of the different classifiers were tested in this paper to obtain a higher performance classifier than the others. The classifiers used are support vector machine (SVM), ensemble, K-nearest neighbors (KNN), linear discriminant analysis (LDA), logistic regression, decision tree, and Naïve bayes. These classifiers were chosen because of their characteristics of high classification speed, small or medium memory usage, which were easy to interpret.

#### Support vector machine (SVM)

In recent years the support vector machine (SVM) classification has become increasingly popular with many applications as a result of its superior performance. The goal of a two-class SVM classifier is to create a hyperplane that maximizes the margin, which is the distance between the nearest points on either side of the boundary. This are known as support vectors. The SVM algorithm can be a linear classifier where the class separation is a straight line, or a nonlinear classifier, where the class separation is a nonlinear line or curve, and a soft-margin formulation where SVM soft-margin formulation may be used in cases where there is no linear hyperplane capable of separating the data [42, 43].

#### Ensemble

Ensemble classifiers incorporate a variety of classifiers to boost the performance of the classification. It is better suitable for multi-class EEG time-varying signal grouping. For the following two factors, the ensemble methods are suitable for the EEG classification. First, the EEG signal dimension is always large and one of preconditions is always to train the classifier as soon as possible, so the training range must also be low. Second, EEG is a time-varying signal, and it is therefore unsafe to use an individual trained classifier to identify the classes of undefined (incoming) objects. Despite these benefits, ensemble studies have failed to achieve a foothold in science and relatively few studies exist in this area [33].

#### K-nearest neighbor (KNN)

K nearest neighbor algorithm is a technique that takes a dummy variable to distinguish the signal in various groups. The result is determined by the number of votes cast by its neighbors, that is one of the several reasons of the its name K-nearest neighbor [34].

#### Linear discriminant analysis (LDA)

Linear discriminant analysis (LDA) is used to locate a particular mix of features that can help distinguish two or more groups. The LDA chooses a path that offers optimum linear class separation. LDA combines objects under equally identical categories on the basis of their characteristics. The purpose of this analysis is to find the appropriate discriminant function which divides classes. If the number of classes is 2 therefore the function has become a line, but when the number of classes is 3 the function would be a plane, for further than 3 classes the discriminant function is a hyperplane. The training set shall be used to determine the parameters of the discriminant function [44].

#### Logistic regression (LR)

Logistic regression (LR) is a commonly applied predictive simulation method that the probability of a dichotomous outcome case is linked to a number of variables. Logistic regression provides fewer strict criteria than ordinary linear regression (OLR) such that it will not presume a linear association between the explanatory variables as well as the response parameter and will not need Gaussian-distributed independent variables. Logistic regression measures the variations in the logarithm of the response variable, instead of the variability of the dependent variable actually, like OLR implies. Although the logarithm of odds is directly proportional to the explanatory variables, the association between the outcome and the explanatory variables would not be linear [38].

#### Decision tree (DT)

The key goal of decision tree (DT) is to integrate the interpretations of the risk level of epilepsy with maximum recognition accuracy. It also has benefits such as ambiguity management, reputation and comprehensibility. These types of trees are widely recommended for post-classification and processing. The basic representation of DT optimization is clarified with the initial statement, *W* = [*P*i, j] as a co-occurrence matrix where (*i*, *j*) is the overall set of items representing the dimensionally decreased values of a specific epoch containing (20 × 16) items [39].

#### Naïve Bayes (NB)

A Naive Bayes (NB) is a probabilistic algorithm that is dependent on Bayesian theory and claims that each function of a given class is exclusive than some other function. Occurrence/specific omission projections for the NB method are determined by high chance. The NB algorithm needs fewer training data in classification [41].

### Cross-validation and testing

The cross-validation step is essential for validating the performance of the learning algorithm. Cross-validation is used to rate the performance of the classifier by dividing the full data set into a training set and a test set. The classifier is trained by the training set, and the trained model is then tested by the test set. K-fold cross-validation is one of the most common methods used by dividing the dataset into K equal size subsets. K − 1 folds are trained for each validation, and the remaining fold is used for testing. The procedure is going to loop K times. At each iteration, a different subset will be chosen as the new test set to ensure that all samples are included at least once in the test set. If *K* equals the size of the training set, so at each validation run, only one sample is left out; therefore, it is called cross-validation (loocv). The proposed algorithm uses the K-fold method with five subsamples [45, 46].

### Evaluation metrics parameters

This section presents the metric parameters that will be used to measure the performance of the classifier. Each classifier is tested using 30 samples (containing both seizures and normal samples). To test the results, the true positive, the true negative, the false positive and the false negative are defined as [47]:

- *True positive *(*TP*): positive (patient) samples correctly classified as positive (patient) samples.
- *False positive* (*FP*): negative (normal) samples incorrectly classified as positive (patient) samples.
- *True negative* (*TN*): negative (normal) samples correctly classified as negative (normal) samples.
- *False negative* (*FN*): positive (patient) samples incorrectly classified as negative (normal) samples.

The parameters TP, TN, FP, and FN will be used to calculate the metric parameters that will be used to measure the performance of the different classifiers. These parameters are: [47, 48]

15 $$\mathrm{Accuracy}= \frac{\mathrm{TP}+\mathrm{TN}}{\mathrm{TP}+\mathrm{FP}+\mathrm{TN}+\mathrm{FN}}\times 100\mathrm{\%}$$

16 $$\mathrm{Pr}=\mathrm{ sensitivity}=\frac{\mathrm{TP}}{\mathrm{TP}+\mathrm{FN}}\times 100$$

17 $$\mathrm{specificity}=\frac{\mathrm{TN}}{\mathrm{TN}+\mathrm{FP}}\times 100\mathrm{\%}$$

18 $$\mathrm{fall}{\text -}\mathrm{Out}=\frac{\mathrm{FP}}{\mathrm{TN}+\mathrm{FP}}$$

19 $$\mathrm{Miss}{\text -}\mathrm{Rate}=\frac{\mathrm{FN}}{\mathrm{TP}+\mathrm{FN}}$$

20 $$\mathrm{positive\,predictivity}=\frac{\mathrm{TP}}{\mathrm{TP}+\mathrm{FP}}$$

21 $$\mathrm{Recall}=\frac{{T}_{P}}{\left({T}_{P}+{F}_{N}\right)}$$

22 $$\mathrm{Precision}=\frac{{T}_{P}}{\left({T}_{P}+{F}_{p}\right)}$$

23 $$F{\text -}{\rm Measure}=2 \times \frac{(\mathrm{Precision }\times \mathrm{ Recall})}{(\mathrm{Precision }+\mathrm{ Recall})}$$

## Data set description

The database used in this study is the CHB-MIT EEG dataset. The CHB-MIT EEG scalp database was collected at the Boston Children’s Hospital in December 2010. The data set consists of 23 cases in 22 patients. The dataset includes five adult males between three and 22 years of age and 17 females between 1.5 and 19 years of age. The dataset has a sampling rate of 256 samples per second at 16-bit resolution [9, 49].

## Experimental results

The model presented consists of two steps. The first step is to train the classifier, and the second step is to test. In the first step, 250 samples were used to train all seven classifiers.

The seven classifiers were tested using two methods during the test phase. The first method that classifiers were tested using a randomized dataset that was taken from several patients. It was specified as 80 samples per sample of 10 s. The proposed algorithm takes only the window of 10 s to speed up the prediction process and improve the accuracy of the detection process instead of taking the whole period for seizure and normal EEG signal. In addition, to reduce the features as the whole EEG signal has several features than taking only 10 s.

The second test method is a continuous dataset test, which has been taken from only one patient at different times. The data indicated that 82 samples were taken from only one patient in a variety of seizures and normal events, such as:

- If Seizure event starts from 2996 to 3026 s, a short period of 10 s will be taken from 2996 to 3006, 3006–3016, and 3016–3026) these are three samples.
- If Seizure event starts from 1862 to 1902s, a short period of 10 s will be taken from 1862–1872, 1872–1882, 1882–1892 and 1892–1902) these are four samples. And so on.

The algorithm proposed consists of five steps, as described above. The first step is the selection of the channel, then the extraction of the features; the third step is the average, the fourth step is the cross-validation and, finally, the classification step.

### Variance channel selection

The sample of 10 s consists of 23 channels. The variance parameter will be calculated for each channel. Only three channels with the highest variance parameter will be selected in this step. Only one sample, including 23 channels, is shown in Table 1 and the variance parameter for each sample has been calculated. The three highest variance parameters are for channels 2, 6, and 21. The selected channels will be on channels 2, 6 and 21. The same step is applied to all other samples.

**Table 1:** Calculation of the variance for the 23 channels and channel selections

CHn	VAR	CHn	VAR	CHn	VAR
CH1	1.44E + 05	CH9	7.23E + 04	CH17	7.22E + 04
*CH2*	*2.14E + 05*	CH10	4.09E + 04	CH18	6.64E + 04
CH3	9.76E + 04	CH11	2.97E + 04	CH19	9.76E + 04
CH4	8.92E + 04	CH12	4.10E + 04	CH20	7.76E + 04
CH5	1.37E + 05	CH13	8.32E + 04	*CH21*	*1.56E + 05*
*CH6*	*1.51E + 05*	CH14	4.79E + 04	CH22	2.02E + 04
CH7	4.16E + 04	CH15	8.25E + 04	CH23	8.25E + 04
CH8	7.67E + 04	CH16	3.62E + 04		

Italic values indicate the three highest variance parameters are for channels 2, 6, and 21 that will be selected for next step

### Feature extraction and averaging

After selecting the three highest channels, the features will be extracted from these three channels. Eleven features with standard deviation, mean, variance, median, kurtosis, skewness, entropy, moment, power, maximum and minimum EEG signals will be extracted from the three channels. Table 2 shows the extracted features of the three channels selected from the previous step. The same step shall be applied to all other samples.

**Table 2:** Extracted features of selected channels

STD	Mean	Max	Min	Var	Med	SKW	ENT	KRT	MOM	POW	CHn
463.10	1.61	1095.00	− 1385.00	2.14E + 05	17.00	− 0.11	4.70	2.35	1.08E + 11	1.41E + 12	CH2
395.41	9.95	1279.00	− 1295.00	1.56E + 05	− 25.00	0.26	4.47	3.00	7.33E + 10	1.03E + 12	CH21
388.61	4.13	941.00	− 1293.00	1.51E + 05	42.00	− 0.40	4.84	3.01	6.86E + 10	9.90E + 11	CH6

The extracted features of the three channels will be averaged into only one value per each feature. This proposed method was used to reduce the number of features in each sample. So, each sample will be represented with only 11 features than 33 features in the preceding step. Table 3 presents the averaging step of the previous sample shown in Table 2.

**Table 3:** Averaged extracted features of the three selected channels

STD	Mean	Max	Min	Var	Med	SKW	ENT	KRT	MOM	POW
415.71	5.23	1105.00	− 1324.33	1.74E + 05	11.33	− 0.08	4.67	2.79	8.34E + 10	1.14E + 12

The same step will be applied to all other samples. So, each sample will be represented by only 11 features. This step had been applied to the training samples and also the testing samples. First, 250 samples will be used for the training of the seven classifiers. Some of these training samples are shown in Table 4.

**Table 4:** Some of the training samples after averaging step

STD	Mean	Max	Min	Var	MED	SKW	ENT	KRT	MOM	POW	State
415.7081	5.229598	1105	− 1324.33	173,944.2	11.33333	− 0.08272	4.669139	2.788487	8.34E + 10	1.14E + 12	s
467.4201	− 9.18222	1786.667	− 1309	218,865.1	− 20.3333	0.362146	4.292831	3.875785	1.96E + 11	1.44E + 12	s
366.6224	12.58714	1046.333	− 1296.67	134,524.1	17	− 0.21187	4.648266	3.686893	6.7E + 10	8.83E + 11	s
241.3414	− 1.70558	1694.333	− 967	59,063.96	− 2.33333	1.505989	3.675731	15.32168	6.66E + 10	3.87E + 11	n
254.11	− 3.04178	1351.333	− 1346.33	65,207.83	1.666667	− 0.10194	4.134625	7.827703	3.14E + 10	4.28E + 11	n
303.9874	0.370949	1477.667	− 1665	92,876.37	1.333333	0.2845	4.067168	7.142509	6.06E + 10	6.09E + 11	n
311.1377	− 0.80893	1654.667	− 1582	97,136.49	1.666667	− 0.33156	3.97244	8.298503	8.07E + 10	6.37E + 11	n
347.6363	− 3.59677	955	− 1061	123,772.3	5	0.019804	4.49983	3.803735	6.33E + 10	8.12E + 11	s
277.2208	0.704282	841.6667	− 798.667	76,893.41	− 1.66667	0.175046	4.582805	3.129974	1.87E + 10	5.04E + 11	s

### Classification

The extracted and averaged features will be used as an input to the seven classifiers that are support vector machine (SVM), ensemble, K-nearest neighbors (KNN), linear discriminant analysis (LDA), logistic regression, decision tree, and Naïve Bayes. The performance of each classifier will be calculated by measuring several performance metric parameters.

### Cross-validation and testing

In this step, the data will be divided into training and testing sets. Two hundred and fifty samples will be used to train all classifiers. The testing will be carried out using two methods, random and continuous.

**Fig. 2:**
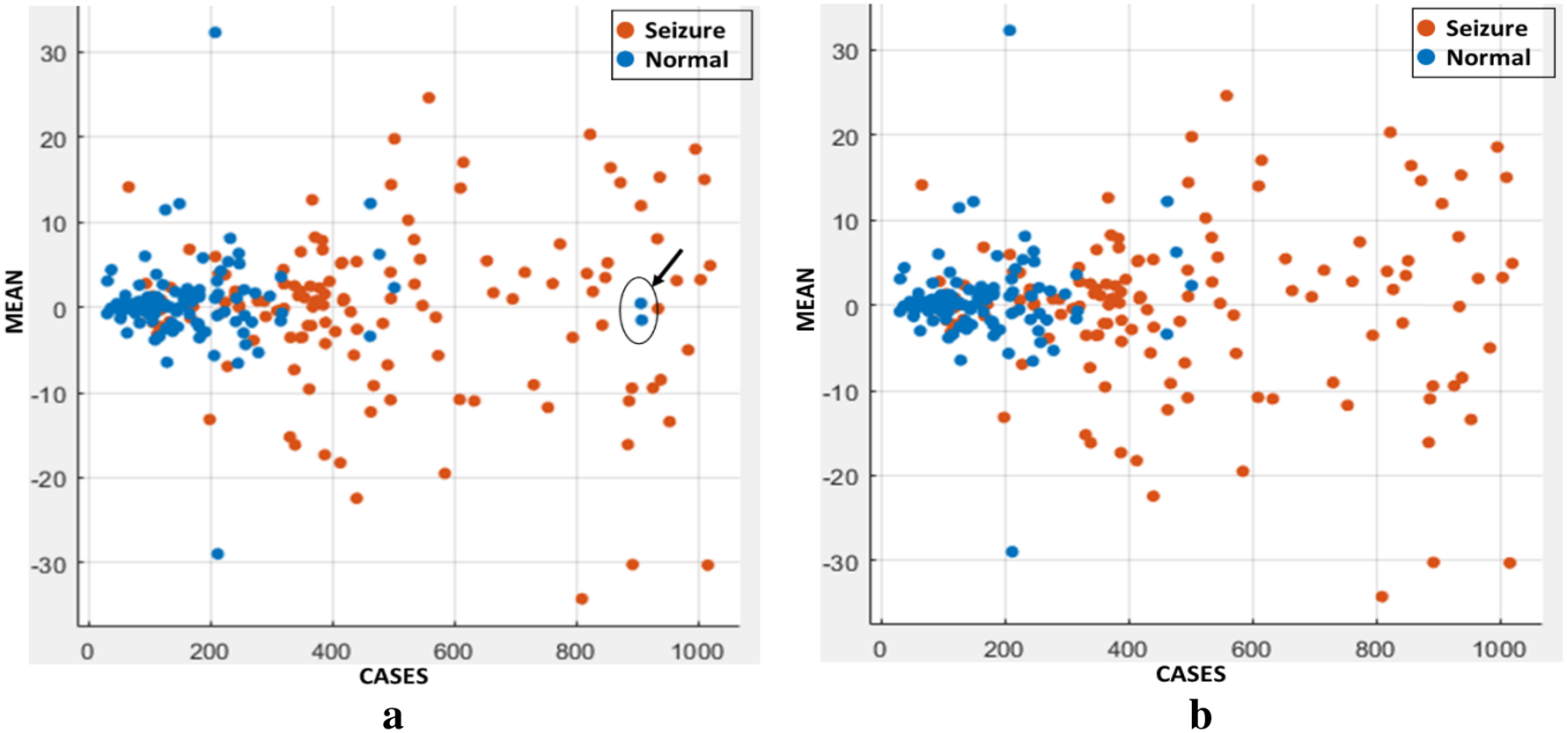
Dataset distribution **a** dataset with abnormal points, **b** dataset without abnormal points

A vital data observation step should be performed before starting the training of classifiers; this will give us an indication of the spread of normal samples and seizure samples—data observation with any of the 11 features. The (mean) feature was chosen to plot this data observation, as shown in Fig. 2.

From Fig. 2a, the normal blue samples are concentrated on the left side, and the red Seizure samples are concentrated on the right side.

Two normal samples were located on the right side between Seizure samples as shown in the arrow in Fig. 2a. These abnormal samples may be accurate samples or sound samples. Figure 2b shows that this is a noise sample and will be removed from training samples. Table 5 shows the accuracy of the seven classifiers (in column 1) for both abnormal samples (column 2) and normal samples (column 3).

**Table 5:** Training results accuracy with filtered and unfiltered samples

Model	Accuracy [un-filtered data]	Accuracy [filtered data]
SVM	81.3	83.2
Ensemble	80.1	82.8
KNN	83.3	84.8
LDA	81.3	82.8
Logistic regression	80.9	84.8
Decision tree	78.1	82.4
Naïve Bayes	75.2	80.4

After training, the seven classifiers will be tested using two methods: random and continuous test sets. The randomized test data set consists of different patients who are not connected to the EEG signal; this means that the samples taken are from different periods of time.

**Fig. 3:**
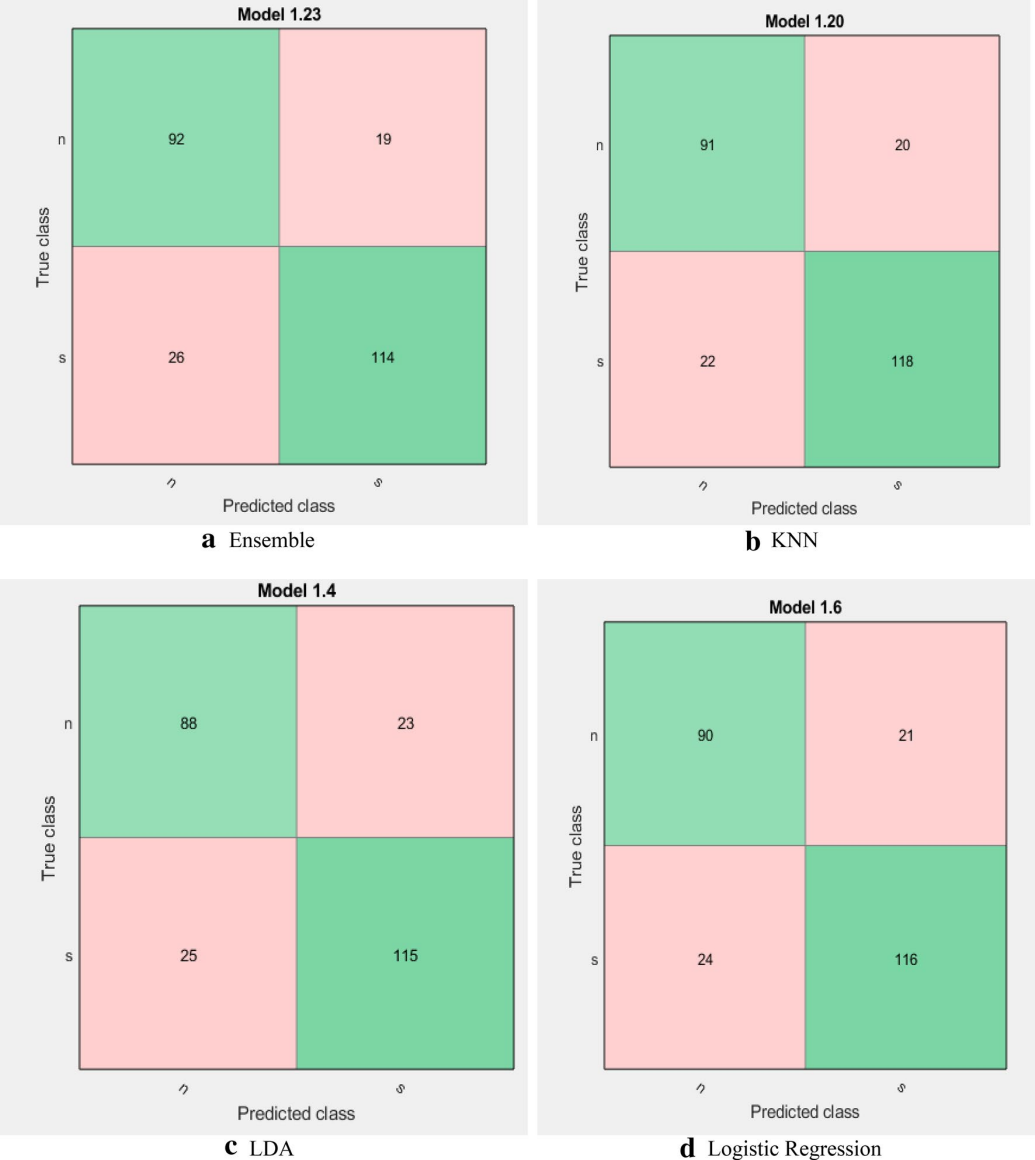

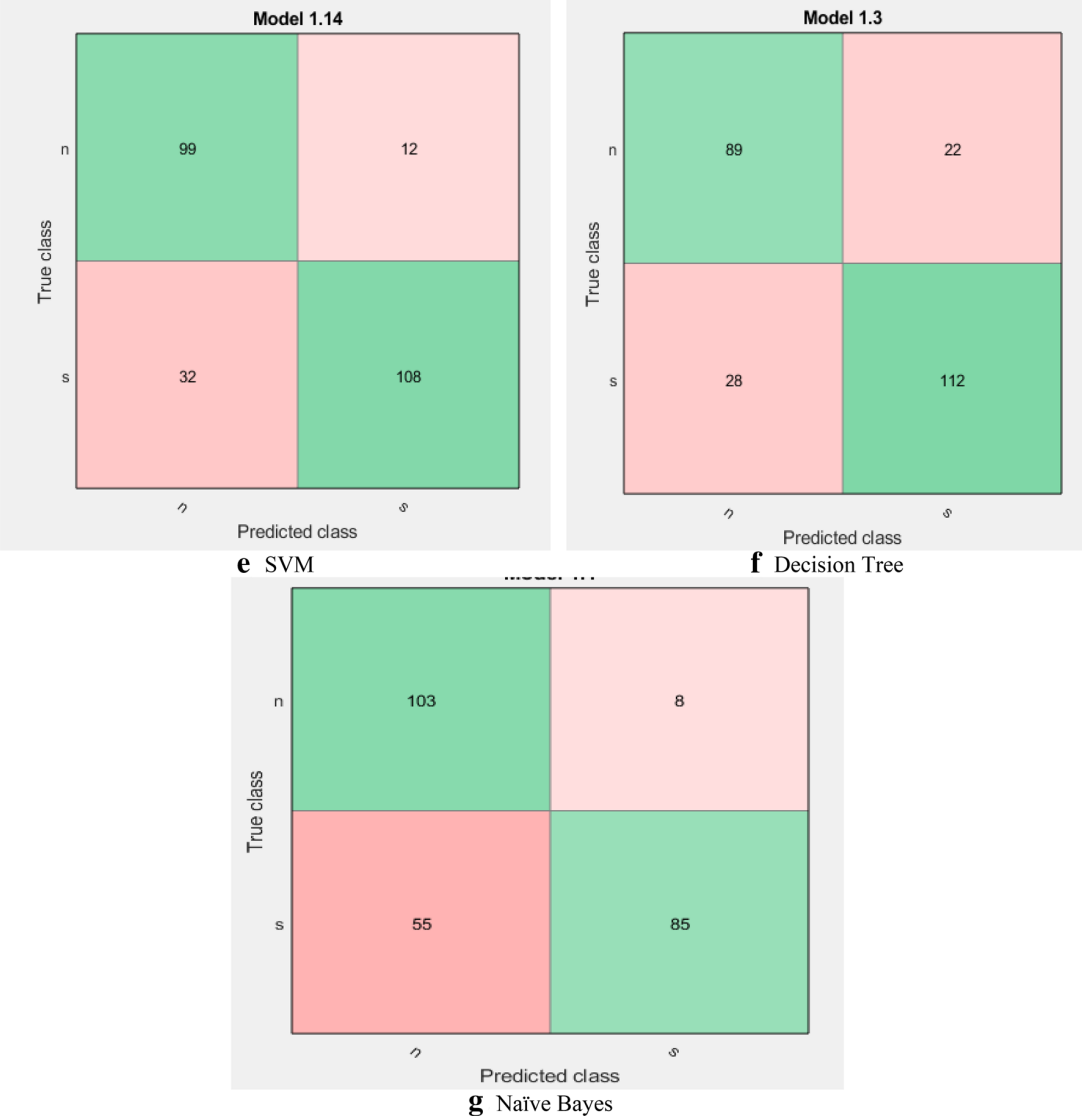
Confusion matrices for all classifiers

Figure 3 shows the confusion matrix for each classifier. The *x*-axis presents the predicted classes by the classifier and the *y*-axis is the true values. The confusion matrix is essential for the representation of the samples. For example, the KNN classifier truly predicted 118 samples as seizure and incorrectly predicted 20 samples as normal. In addition, the KNN truly predicted 91 samples as normal and incorrectly predicted 22 samples as a seizure. All confusion matrices of all classifiers are presented in Fig. 3.

**Fig. 4:**
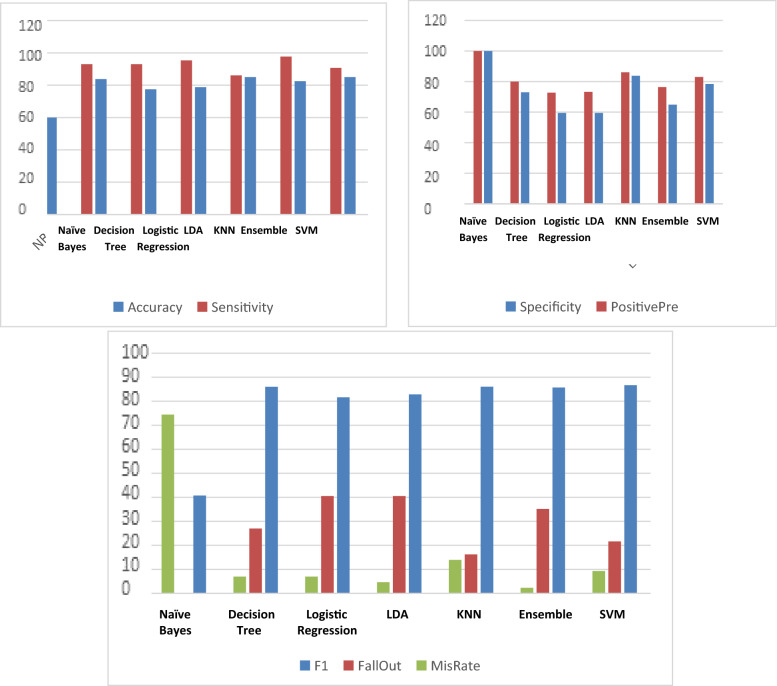
Evaluation of the metric parameters for the seven classifiers of the random case testing method

Table 6 and Fig. 4 present 11 metric evaluation parameters, which are true positive (TP), true negative (TN), false positive (FP), false negative (FN), accuracy, sensitivity, specificity, positive predictivity, F1 score, Fall-Out, and Mis-Rate, which were measured by all seven classifiers.

**Table 6:** Evaluation of the metric parameters for the seven classifiers of the random case testing method

	SVM	Ensemble	KNN	LDA	Logistic regression	Decision tree	Naïve Bayes
TP	39	42	37	41	40	40	11
TN	29	24	31	22	22	27	37
FP	8	13	6	15	15	10	0
FN	4	1	6	2	3	3	32
Accuracy	85	82.5	85	78.75	77.5	83.75	60
Sensitivity	90.69767	97.67442	86.04651	95.34884	93.02326	93.02326	25.58
Specificity	78.37838	64.86486	83.78378	59.45946	59.45946	72.97297	100
PositivePre	82.97872	76.36364	86.04651	73.21429	72.72727	80	100
F1	86.66667	85.71429	86.04651	82.82828	81.63265	86.02151	40.75
FallOut	21.62162	35.13514	16.21622	40.54054	40.54054	27.02703	0
MisRate	9.302326	2.325581	13.95349	4.651163	6.976744	6.976744	74.42

**Fig. 5:**
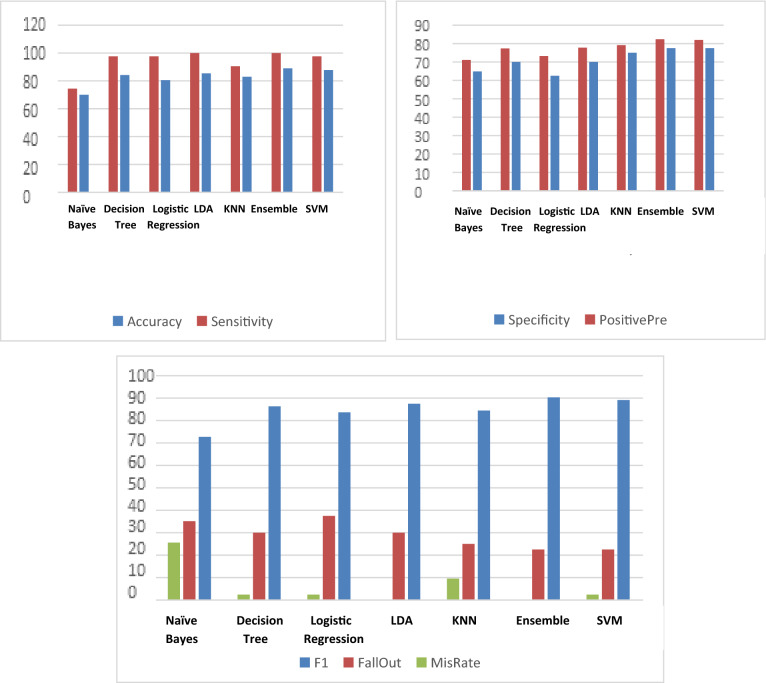
Evaluation of the metric parameters for the seven classifiers of the continuous case testing method

The second testing method is continuous testing data. Continuous means that the test samples are connected to the same person. Data were collected from only one patient with connected EEG signals. Table 7 and Fig. 5 show 11 metric evaluation parameters that were measured for all seven classifiers.

**Table 7:** Evaluation of the metric parameters for the seven classifiers of the continuous case testing method

	SVM	*Ensemble*	KNN	LD	Logistic regression	Decision tree	Naïve Bayes
TP	41	42	38	42	41	41	32
TN	31	31	30	28	25	28	24
FP	9	9	10	12	15	12	13
FN	1	0	4	0	1	1	11
Accuracy	87.80488	89.02439	82.92683	85.36585	80.4878	84.14634	70
Sensitivity	97.61905	100	90.47619	100	97.61905	97.61905	74.42
Specificity	77.5	77.5	75	70	62.5	70	64.86
PositivePre	82	82.35294	79.16667	77.77778	73.21429	77.35849	71.11
F1	89.13043	90.32258	84.44444	87.5	83.67347	86.31579	72.73
FallOut	22.5	22.5	25	30	37.5	30	35.14
MisRate	2.380952	0	9.52381	0	2.380952	2.380952	25.58

**Fig. 6:**
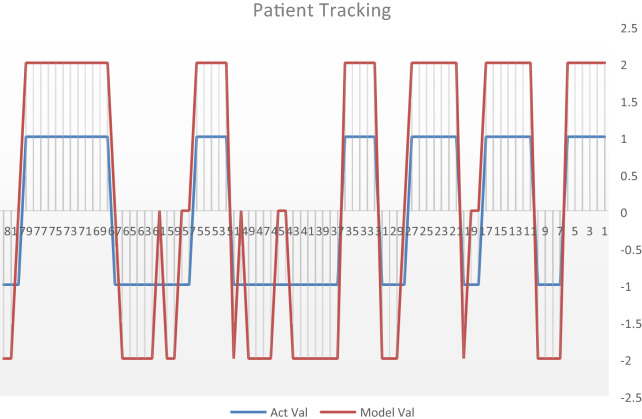
Actual and predicted samples of the ensemble classifier in continuous test

The performance of the classifiers was graphically depicted to display the efficiency and accuracy of each classifier using a continuous test method. Figure 6 displays this graphical representation of the proposed algorithm based on ensemble classifier. In this figure, different samples are evaluated by the ensemble classifier. If the samples are shown above zero, this means that the samples are Seizure and the normal samples are shown below zero. The blue samples are the actual results of the samples and the red samples are the predicted samples of the proposed ensemble classifier approach.

The proposed approach based on the ensemble classifier correctly predicted the samples with numbers 1, 2, 3, 4, 5, 6, 11, 12, 13, 14, 15, 16 and 17 as seizure and they were also actual Seizure. In addition, the proposed approach based on the ensemble classifier correctly predicted the samples with numbers 7, 8, 9, 10, and 20 as normal and they were also normal. The ensemble incorrectly predicted that the two samples with numbers 18 and 19 as a seizure sample, but they were normal. Figure 6 shows all of the other samples.

## Results and discussion

The results obtained from Tables 6 and 7 and Figs. 4 and 5 present 11 metric evaluation parameters, which are true positive (TP), true negative (TN), false positive (FP), false negative (FN), accuracy, sensitivity, specificity, positive predictivity, F1 score, Fall-Out, and Mis-Rate for seven classifiers that are support vector machine (SVM), ensemble, K-nearest neighbors (KNN), linear discriminant analysis (LDA), logistic regression, decision tree, and Naïve Bayes. Table 6 and Fig. 4 showing the metric evaluation parameters for the seven classifiers in random case testing, the KNN classifier is better than the others in accuracy, specificity, positive predictivity, but the ensemble is better than the others in sensitivity and missing rate. The proposed algorithm based on the KNN classifier has a high rate of error (13.9%) compared to the proposed algorithm based on the ensemble classifier (2.3%). Table 7 and Fig. 5 present the metric evaluation parameters for the proposed algorithm based on each classifier of the seven classifiers in the continuous case test method, the proposed algorithm based on the Ensemble classifier is better than the proposed algorithm based on other classifiers in all metric parameters. Figure 6 shows that the proposed algorithm based on Ensemble classifier detected all seizure cases without any error, but there is some difficulty in detecting all normal cases.

## Conclusion

This paper proposed a computer aided seizure diagnosis classification system based on feature extraction and channel selection using EEG signals. The proposed approach is evaluated through different experiment circumstances over CHB-MIT dataset. This proposed approach is based on five steps. The first step is to select a channel by calculating the variance parameter for each channel, as each sample consists of 23 channels. The highest three channels of variance will be selected. The second step was to extract eleven features from the selected three channels then averaging these extracted features of the three channels to only one value per feature. As a result, each sample will be represented as only 11 features. The third step is classification, where seven classifiers had been used and experimented these classifiers are support vector machine (SVM), ensemble, K-nearest neighbors (KNN), linear discriminant analysis (LDA), logistic regression, decision tree, and Naïve Bayes. The fourth step is cross-validation and testing, as the data is divided into five sets of training and testing sets. The training set consisted of 250 samples, each of which will be represented by 11 features generated from the first two steps. The testing was carried out using two different methods; the first was randomized case testing, which the EEG samples had been collected from different patients and the second was continuous case testing method that the EEG samples had been collected from only one patient. The last step is the evaluation of the classifiers and measuring the performance of the classifiers. In the first randomized case testing method, the proposed approach based on the KNN classifier is better than the other classifiers in accuracy, specificity, positive predictivity, but the proposed approach based on the ensemble is better in other metric parameters such as sensitivity and missing rate. The KNN has a high rate of error (13.9%) compared to the ensemble (2.3%). In the second continuous case testing method. The proposed approach based on the ensemble classifier is better classified than the other classifiers in all metric parameters,

In future work, the proposed approach based on the ensemble classifier predicted all seizure cases without any error, but there is some difficulty in predicting all normal cases that will be recovered in the future then compared with several previous algorithms. In addition, IoT system will be proposed which is based cloud framework for collect, store, and analyze data from patient wearable devices with the scalability to millions of users. Finally, the proposed approach based on deep learning will be proposed for accurate detection of seizures over different available datasets.
